# Detection and Identification of the Atypical Bovine Pestiviruses in Commercial Foetal Bovine Serum Batches

**DOI:** 10.1371/journal.pone.0028553

**Published:** 2011-12-08

**Authors:** Hongyan Xia, Balaje Vijayaraghavan, Sándor Belák, Lihong Liu

**Affiliations:** 1 Department of Biomedical Sciences and Veterinary Public Health, Swedish University of Agricultural Sciences, Uppsala, Sweden; 2 Department of Virology, Immunobiology and Parasitology, National Veterinary Institute, Uppsala, Sweden; University of Kansas Medical Center, United States of America

## Abstract

The recently emerging atypical bovine pestiviruses have been detected in commercial foetal bovine serum (FBS) of mainly South American origin so far. It is unclear how widely the viruses are presented in commercial FBS of different geographic origins. To further investigate the possible pestivirus contamination of commercially available FBS batches, 33 batches of FBS were obtained from ten suppliers and analysed in this study for the presence of both the recognised and the atypical bovine pestiviruses. All 33 batches of FBS were positive by real-time RT-PCR assays for at least one species of bovine pestiviruses. According to the certificate of analysis that the suppliers claimed for each batch of FBS, BVDV-1 was detected in all 11 countries and BVDV-2 was detected exclusively in the America Continent. The atypical pestiviruses were detected in 13 batches claimed to originate from five countries. Analysis of partial 5′UTR sequences showed a high similarity among these atypical bovine pestiviruses. This study has demonstrated, for the first time that commercial FBS batches of different geographic origins are contaminated not only with the recognised species BVDV-1 and BVDV-2, but also with the emerging atypical bovine pestiviruses.

## Introduction

The genus *Pestivirus* consists of four recognized species: *Bovine viral diarrhoea virus* 1 (BVDV-1), *Bovine viral diarrhoea virus* 2 (BVDV-2), *Border disease virus* (BDV) and *Classical swine fever virus* (CSFV); and a tentative species, Pestivirus of giraffe [1]. Pestivirus can cross placenta and infect foetuses, which may be aborted or persistently infected after birth. Foetal bovine serum (FBS) has been found contaminated with bovine pestiviruses since 1960s. Recently, atypical bovine pestiviruses were detected in bovine samples collected in different regions of the world, such as commercial FBS of South American origin and bovine serum samples originating from Thailand. These viruses include D32/00_‘HoBi’ [2], CH-KaHo/cont [3], SVA/cont-09 [4], and IZSPLV_To [5], two strains in aborted foetuses in Brazil [6], Brz buf 9 in a Brazilian buffalo [3], Th/04_KhonKaen in a calf in Thailand [7], [8], [9], and Italy-1/10-1 associated with an outbreak of severe respiratory disease in Italy [10]. Phylogenetic analysis has revealed a sister relation to BVDV-1 and BVDV-2 with strong support, and BVDV-3 has been proposed to represent this new bovine pestivirus species [11]. Due to genetic variation, the so-called pan-pestivirus primers 324/326 [12] may fail to detect these viruses [2], [5]; therefore a new real-time TaqMan assay has been developed [13]. By this assay, a batch of FBS that was claimed to be of Australian origin was found positive for the new atypical pestivirus, indicating probably a much wider distribution than previously thought (unpublished). The objective of this study was to systematically investigate, by real-time RT-PCR and DNA sequencing, the presence of both the recognised species (BVDV-1 and BVDV-2) and the newly described atypical bovine pestiviruses in commercial FBS batches from major producers.

## Results and Discussion

All 33 batches of FBS were found containing at least one species of bovine pestiviruses. Twenty-nine batches were found positive for BVDV-1, 11 batches positive for BVDV-2, and 13 batches positive for the atypical bovine pestivirus (Table 1). According to the country of origin that the producers claimed for each batch of FBS, BVDV-1 was detected in all 11 countries and BVDV-2 was detected exclusively in the America Continent. The atypical bovine pestiviruses were detected in FBS batches claimed to be of American, Australian, Brazilian, Canadian, and Mexican origin. Presence of the atypical bovine pestiviruses in Brazil was demonstrated in three batches of FBS from three suppliers, indicating that the viruses may have been spread widely in the country. This is in agreement to previous observation that several strains were found in FBS of Brazilian origin [2], [14]. The viruses were also found in five batches of FBS of Australian origin from three suppliers (B, G, and H), but not from the supplier A. Similarly, the atypical pestiviruses were detected in two batches of USA origin from two suppliers (B and H) but was negative in seven batches from three suppliers (A, C and E). Presence of the atypical bovine pestiviruses in Canada and Mexico was demonstrated in one of three batches. One batch was also positive, but it was only identified as South American origin. The examined FBS batches originating from Colombia, Denmark, Dominican Republic, France, New Zealand, South Africa and two unidentified countries were negative for the atypical bovine pestiviruses.

**Table 1:** The batches of foetal bovine serum tested by real-time RT-PCR.

Supplier	Sample ID	Origin	BVDV-1	BVDV-2	BVDV-3
A	A1	Australia	+	−	−
	A2	Brazil	−	−	+
	A3	USA	+	+	−
	A4	USA	+	+	−
	A5	USA	+	+(ns)	−
B	B1	Australia	+	−	+
	B2	Australia	+	−	+
	B3	Australia	−	−	+
	B4	Canada	+	+(ns)	+
	B5	Mexico	+	+	+
	B6	USA	+(ns)^a^	−	+
C	C1	USA	+	−	−
	C2	USA	+	−	−
D	D1	USA	+	+	−
E	E1	Canada	+	+	−
	E2	EU	+	−	−
	E3	New Zealand	+	−	−
	E4	South American	+	−	+
	E5	USA	+	+	−
F	F1	Brazil	+	+	+
G	G1	Australia	−	−	+
	G2	Brazil	−	−	+
H	H1	Australia	+	−	+
	H2	Mexico	+	+(ns)	−
	H3	USA	+	−	+
J	J1	South Africa	+	−	−
K	K1	Canada	+	+	−
	K2	Colombia	+	−	−
	K3	Denmark	+	−	−
	K4	Dominican Republic	+	−	−
	K5	France	+	−	−
	K6	Mexico	+	−	−
	K7	Unidentified	+	−	−

a Sequence has not been determined.

**Figure 1:**
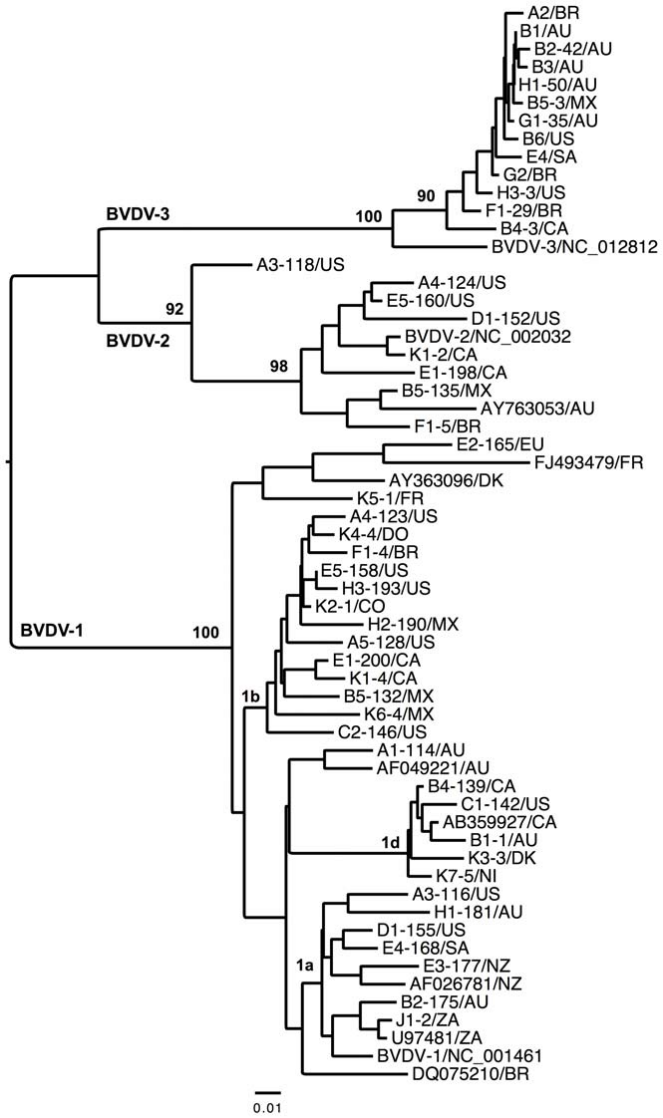
Neighbor-Joining analysis of a 249-bp fragment of the 5′UTR sequences under Kimura 3-parameter model. The sequences are labelled with sample ID (-number of clone)/country or region of origin. AU, Australia; BR, Brazil; CA, Canada; CO, Colombia; DK, Denmark; DR, Dominican Republic; EU, European Union; FR, France; MX, Mexico; NI, not identified by supplier; NZ, New Zealand; SA, South American; US, USA; ZA, South Africa. Numbers are percentage of bootstrap values (1000 replicates) for major clades. Bar indicates changes per site. The GenBank accession numbers for reference strains are DQ075210, NC_001461, U97481, AF026781, AB359927, AF049221, AY363096 and FJ493479 for BVDV-1, AY763053 and NC_002032 for BVDV-2, and NC_012812 for BVDV-3.

The newly determined sequences have been deposited in GenBank with accession numbers JN967700–JN967748. By studying the genetic relationship of the detected BVDV-1 and BVDV-2 viruses, the neighbour-joining analysis of 49 partial 5′UTR sequences revealed a certain grouping, but no particular clustering pattern in general in relation to the country of origin (Figure 1), with the exception of clustering of two sequences from the same countries. On the other hand, all sequences of the atypical pestivirus species formed a well-supported clade, which is in agreement with our previous results [4]. The genetic distance of partial 5′UTR was rather short between the strains of different geographical locations.

There are many technical questions here, which need careful investigations and openness from the side of the producers. For example, a possible scenario is that mixing of raw serum materials of different origin might have occurred at certain companies, intentionally or unintentionally, during manufacturing steps. As companies may collect raw serum materials from different geographic locations and transport the frozen sera to a manufacturing site for further processing, including mixing, heat inactivation, gamma irradiation, sterile filtration, and/or centrifugation. It is a possibility that the facility is not cleaned enough after producing one batch, so a small amount of raw sera collected from one location (e.g. South America) was mixed with sera collected from another location (e.g. Australia), and even such a small amount of residue sera is tested positive by the highly sensitive real-time RT-PCR method. Another possible scenario is that the FBS batches may be mistakenly labelled with country of origin different from its real origin. This could happen but it is unlikely all six suppliers had the same conduct. A further alternative is that spreading of the atypical bovine pestiviruses in the above-mentioned countries could have occurred very recently such that viruses had not evolved into distinct groups. This could explain the high sequence similarity among these atypical pestiviruses. Such a scenario would suggest that it is likely that the atypical bovine pestiviruses have a much wider geographic distribution than previously described. Although the origin and the time of emergence of these novel viruses are unknown, a recent study estimated that the emerging of the atypical bovine pestiviruses (BVDV-3a) might occur very recently, around 50 years ago [11].

This study has demonstrated, for the first time that commercial FBS products of different geographic origins are contaminated not only with BVDV-1 and BVDV-2, but also with the emerging pestiviruses. Attempt to isolate bovine pestiviruses from other batches of FBS had been unsuccessful, even though these batches were positive for the presence of BVDV E^rns^ protein by a commercial Antigen ELISA test. Nevertheless, the possible role and importance of the bovine pestiviruses should be thoroughly investigated from several aspects, including veterinary and human medicine and the safety of biological products, such as cell lines and vaccines. In addition, this study also has important implications in the possible problems of pestivirus contamination in research and in diagnostic laboratories, and effect of the emerging pestiviruses on the BVD control programs that are currently implemented in several countries. The occurrence of a severe respiratory disease in Italy caused by the atypical bovine pestiviruses [10], is further emphasizing the importance of the questions raised here. Furthermore, the criteria for determining the risk level of countries producing FBS should be reviewed, with the consideration of the freedom of both the recognised and the atypical bovine pestiviruses. FBS producers should also evaluate the tests for their ability and efficacy to detect pestivirus contamination in raw materials and also in final products. More studies are needed in order to better understand the biological aspects of the atypical bovine pestiviruses and to investigate their hosts, geographical distribution and the disease that caused by the atypical bovine pestiviruses.

## Materials and Methods

A total of 33 batches of FBS were purchased from ten commercial suppliers (Table 1). RNA was extracted from 250 µl of the serum with TRIzol reagent (Invitrogen Corporation, Carlsbad, California), and the pellet was resuspended in 50 µl of water. Real-time RT-PCR was performed as previously described [13] with minor modifications. In brief, the 25-µl reaction mixture contained 5 µl of RNA, 0.6 µM of each primer T134-F and T220-R, 0.1 µM of the probe T155-P, 12.5 µl of 2× buffer, and 0.25 µl of enzyme mix (Qiagen, Hilden, Germany). A duplex real-time RT-PCR assay was performed for the detection of BVDV-1 and BVDV-2 [15]. Both assays were run in parallel on a RotorGene 3000 instrument (Corbett Research, Sydney, Australia). To verify the results, a 258-bp fragment of 5′-end untranslated region (5′UTR) was amplified using a one-step RT-PCR kit (Qiagen, Hilden, Germany), gel-purified and cloned in a vector pCR4-TOPO (Invitrogen Corporation, Carlsbad, California), as previously described [4]. Two published primers Pesti-F and Pesti-R [16] were used for PCR amplification of BVDV-1 and BVDV-2. Plasmid DNA was isolated from overnight culture and sequenced by Macrogen Inc. (Seoul, Korea). Sequence reads were assembled using SeqMan software within Lasergene package (DNASTAR Inc., Madison, WI), and individual pestivirus species was identified by nucleotide blast search in GenBank. Multiple sequence alignment was done using program MAFFT [17] implemented within Seaview (version 4.2) [18]. Neighbor-joining analysis was performed using PAUP* 4.0 [19] under Kimura 3-parameter model [20].
